# SfN 2025 Report: JUNE and FUN Faculty Awards

**DOI:** 10.59390/001c.154876

**Published:** 2025-12-30

**Authors:** Yuan Yuan Kang, Elaine R Reynolds, Jacqueline K Rose

**Affiliations:** 1 Natural Sciences Department University of Houston - Downtown; 2 Biology Department and Neuroscience Program Lafayette College; 3 Psychology Department Western Washington University

**Keywords:** editorial, sfn, faculty awards

The Faculty for Undergraduate Neuroscience (FUN) engaged in many ways at the 2025 Society for Neuroscience meeting in San Diego. Many members attended with their students and colleagues. Saturday night was the FUN Undergraduate Poster Session, in conjunction with the SfN’s Early Career Poster Session, where students and their mentors presented over 100 posters. Seventeen travel awards were awarded. The FUN poster session is always a highlight of the meeting, as we get to see our work with our students on display and watch them transition into young scientists.

Once again, we had a FUN exhibitor booth, which gave our members a place to meet and discuss important issues and SfN attendees a chance to purchase special FUN gifts to take home. A series of talks presented by and for FUN members at the booth covered a wide range of topics, from applying for a faculty position to academic leadership.

Another highlight was the FUN Social and Awards ceremony (see below for a summary of the FUN awards). This year’s social was held on the Monday evening of SfN. at East Village Brewing in San Diego. This event was very well attended by students, faculty, and travel award sponsors. A great time was had by all.

Right after the conclusion of SfN, FUN held the 2025 election with a slate of new officers, including:

**President:** Lorenz Neuwirth

**Past President:** Jackie Rose

**President-elect:** Andreas Nicholas

**Secretary:** Christelle Sabatier

**Treasurer:** Lauren (Rudy) Rudolph

**Treasurer-Elect:** Erica Tracey

**Councilor:** Mathew Abrams, Jennifer Wenzel, Lauren Williamson, Aparna Shah, Jennifer Honeycutt, Thomas Newpher

We thank past officers for their service to FUN.

The FUN Annual Business meeting will be held on Thursday, January 15^th,^ on Zoom this year. A meeting registration link will be sent to members.

## JUNE Awards

JUNE presented several awards to recognize and thank authors, board members, and reviewers who make the journal strong. Implementing DOIs has allowed us to track views, and so we are incorporating that data into our selection of paper of the year. One thing that is striking about

this data is that a paper doesn’t have to be new to gain a readership. Two recent papers (2024) were repeated at the top of the monthly summaries, and the editor and reviewers agreed that these were excellent contributions to the journal. These are:

Memphis NeuroSTART Program: Promoting Student Success and Increasing the Diversity of Applicants to Neuroscience Graduate Programs

By Helen J.K. Sable & Deranda B. Lester

Using BioRender for Active Learning: Exploring Learning-Style Preference and Visual-Spatial Ability in Undergraduate Students

By Jessica Ha, Deena Afana, Keon Nassimi Moghaddam, Andrea Nicholas

We want to thank the editorial staff that has been instrumental in paper review and the process of moving the journal to a new format and website. We are still in the middle of the process, and we thank everyone for your support and patience. The Fall JUNE issue will be the first published on the new website.

Reviewer of the Year is Ian Harrington, Professor at Augustana College. Ian has been on the editorial board for a long time and has led several initiatives, including Amazing Papers and Project Divine. He always says yes! And has been supportive of all of the efforts to improve the journal over the years. During the last year, Ian has been a very active reviewer, especially with the amazing paper series, which is an important contribution to teaching with the primary literature.

## FUN Faculty Awards for 2025

Each year, FUN opens a call for nominations to recognize outstanding contributions to undergraduate education in neuroscience. These awards are presented to FUN members who have exhibited exceptional teaching or research in an undergraduate setting, who have provided fundamental service to advancing the mission and values of FUN, and who have demonstrated achievements in these areas over sustained periods of time. Nominations are solicited and reviewed by the Awards committee, which this year consisted of Yuan Yuan Kang (Past President); Jackie Rose (President); Lauren Williamson (Councilor); Thomas Newpher (FUN member) and Tari Tan (FUN member).

**CAROL ANN PAUL EDUCATOR OF THE YEAR:** This year’s award recipient is Dr. Maureen Rutherford, an Associate Professor in Psychology at Indiana University Northwest (IUN). She is also the Psychology Department Chair and an Associate Director of FACET (Faculty Academy on Excellence in Teaching) at IUN. A winner of the 2022 Indiana University Northwest Founder’s Day Teaching Award, Dr. Rutherford is recognized for her innovations in neuroscience education and support to the Psychology students at her institution, as the faculty advisor for the Psi Chi Honor Society, and for the Psychology and Brain Sciences Club. Her notable pedagogical efforts included developing a neuroscience Course-based Undergraduate Research Experience (CURE) for first-year students and incorporating service-learning projects as part of curriculum and expansion into neuroscience outreach opportunities. In addition to her neuroscience research with zebrafish, Dr. Rutherford has also published in education journals, including *Journal of the Scholarship of Teaching and Learning, Advances in Methods and Practices in Psychological Science,* and *Teaching of Psychology*.

**FUN MENTOR AWARD:** This year’s recipient of the FUN Mentor Award is Dr. David Jewett, a Professor of Psychology and Neuroscience at the University of Wisconsin, Eau Claire. Dr. Jewett is chosen for this award based on his extensive record of supporting trainees at all career stages, ranging from undergraduate students to early career neuroscience faculty. Dr. Jewett’s research team is composed exclusively of undergraduates (typically 10 – 15+ members). He has mentored more than 250 undergraduates in research, with most of them having presented their work at regional and national conferences and many of them continuing to PhD graduate training and research-related careers. Dr. Jewett has also mentored students in the MARC (minority access to research careers) programs and through the Ronald E McNair program that supports talented low-income, first-generation students. Dr. Jewett supports and has co-hosted the Midbrains regional undergraduate neuroscience conference. Beyond undergraduate students, Dr. Jewett supports curriculum for and personally mentors trainees across the career spectrum in their scientific development. A notable example is the development of the FUN Mentoring Network, an in-person program to support faculty who engage undergraduates in neuroscience classrooms and laboratories at all levels, and in a diversity of careers and institutions.

**FUN SERVICE AWARD:** This year’s service award goes to Dr. Elaine Reynolds for her extraordinary leadership in stewarding JUNE through its modernization and restructuring process as the Editor-in-Chief. In her three-year term (2022-2025), Elaine formalized the operational procedure of JUNE and restructured our copyediting staff and editorial board. With Bill Grisham, she tirelessly assigned hundreds more digital object identifiers (DOIs) to *JUNE* articles and assured that these were indexed in PubMed. Above all, Dr. Reynolds oversaw key changes to modernize and advocate for JUNE as the flagship FUN journal. Specifically, Dr. Reynolds researched and identified Scholastica as the new platform for hosting JUNE, which included moving JUNE operations to Scholastica, while working with all stakeholders to ensure its smooth transition, all of which have taken many hours, emotional labor, and technical troubleshooting. In the end, these changes will have a long-term positive impact on our journal, whether in giving legitimacy in the eyes of many other scientists or in ensuring sustainability and consistent support for the journal.

**CAREER AND LIFETIME ACHIEVEMENT AWARD:** Dr. Elaine Reynolds, Professor in Biology and Neuroscience at Lafayette College, is the recipient of this year’s Career and Lifetime Achievement Award, for showing continuous care for the undergraduate neuroscience community and its advancement toward progressive goals for the past 25 years. During this time, Dr. Reynolds mentored 71 conference presentations and published 17 research articles with undergraduate neuroscience students. As the founding member, President, and Vice President for her regional chapter of the Society for Neuroscience, Dr. Reynolds won multiple teaching and mentoring awards, including the 2016 Carol Anne Paul Educator of the Year Award and 3 separate awards from Lafayette College in 2002, 2016, and 2018. A long-time FUN member, Dr. Reynolds has served on the Executive Board, as President, and Past President between the years of 1999-2004, continuing her service on the JUNE editorial board since 2015, and eventually taking up the role of Editor-in-Chief in 2022. As Editor-in-Chief of JUNE, Elaine has promoted critical reflection, culture change toward increased inclusion and social justice, and intellectual debate on the impact of a changing technological landscape for undergraduate neuroscience. These priorities are reflected in her contributions to the 2023 FUN Pedagogical Workshop Committee and publication in the JUNE workshop issue. Most recently, she has spearheaded the assembly of an Oxford Handbook of Undergraduate Neuroscience Education, issued a call to action urging the undergraduate neuroscience community to reinvest in public engagement, and put her own advice on this matter into practice through her long-standing outreach activities with her local senior community.

**NEUROSCIENCE CHAMPION AWARD:** Dr. Mays Imad is a Professor in Biology at Connecticut College. Dr. Imad promotes a perspective of deep humanism and recognizes the human experience and worth of every individual as a primary condition for learning. In turn, Dr. Imad has brought this humanistic perspective to critique the structures of academic policies and institutional practices regarding STEM culture. An example of this work can be found in an article she published titled “Recasting the agreements to re-humanize STEM education,” which appeared in a special issue in *Frontiers in Education* under the theme “Centering Humanism in STEM Education”. As this year’s Neuroscience Champion Award recipient, Dr. Imad was not only selected based on her body of work in inclusive excellence, but also for bringing those ideas to the undergraduate neuroscience community and to the benefit of a broad range of STEM educators and their students. Notably, Dr. Imad’s trauma-informed pedagogy delivered at the 2020 Summer Virtual Meeting served as the source of inspiration for FUN faculty with her inimitable emotional authenticity in a moment of profound crisis and social disconnection. At the 2023 FUN workshop at Western Washington University, she delivered a hands-on workshop on “recasting the agreement to re-humanize STEM education” and further extended its intellectual reach to a broader range of education practitioners and researchers through an open panel she organized at the 2023 meeting of the Society for Social Studies of Science (4S) in Honolulu, Hawaii. In 2025, Dr. Imad co-organized a panel at the Society for Social Studies of Science, in Seattle, WA on the theme “Representation, Reclamation, and Epistemological Boundaries in Scientific Narratives”. As stated in her nomination letter, Dr. Imad is “a Neuroscience Champion because she uses her prodigious talents to understand the structural phenomena (economical, political, and social features of systems) that constrain our actions, encourage us to connect with our own deepest moral intentions, connect those intentions to our capacity to act, and build our capacity to act collectively through building our connections with one another.”

### Address correspondence to:

Dr. Elaine R Reynolds, Department of Biology and Program in Neuroscience, Lafayette College, Easton, PA 18042.

Email: reynolde@lafayette.edu

